# Fixed-Bed Column Technique for the Removal of Phosphate from Water Using Leftover Coal

**DOI:** 10.3390/ma14195466

**Published:** 2021-09-22

**Authors:** Dereje Tadesse Mekonnen, Esayas Alemayehu, Bernd Lennartz

**Affiliations:** 1 School of Chemical Engineering, Jimma Institute of Technology, Jimma University, Jimma P.O. Box 378, Ethiopia; getdere@gmail.com; 2 Faculty of Agricultural and Environmental Sciences, University of Rostock, Justus von-Liebig Weg 6, 18059 Rostock, Germany; 3 Faculty of Civil and Environmental Engineering, Jimma Institute of Technology, Jimma University, Jimma P.O. Box 378, Ethiopia; 4 Africa Center of Excellence for Water Management, Addis Ababa University, Addis Ababa P.O. Box 1176, Ethiopia

**Keywords:** breakthrough curve, fixed-bed column, bed height, adsorption, eutrophication

## Abstract

The excessive discharge of phosphate from anthropogenic activities is a primary cause for the eutrophication of aquatic habitats. Several methodologies have been tested for the removal of phosphate from aqueous solutions, and adsorption in a flow-through reactor is an effective mechanism to reduce the nutrient loading of water. This research aimed to investigate the adsorption potential of leftover coal material to remove phosphate from a solution by using continuous flow fixed-bed column, and analyzes the obtained breakthrough curves. A series of column tests were performed to determine the phosphorus breakthrough characteristics by varying operational design parameters such as adsorbent bed height (5 to 8 cm), influent phosphate concentration (10–25 mg/L), and influent flow rate (1–2 mL/min). The amorphous and crystalline property of leftover coal material was studied using XRD technology. The FT-IR spectrum confirmed the interaction of adsorption sites with phosphate ions. Breakthrough time decreased with increasing flow rate and influent phosphate concentration, but increased with increasing adsorbent bed height. Breakthrough-curve analysis showed that phosphate adsorption onto the leftover coal material was most effective at a flow rate of 1 mL/min, influent phosphate concentration of 25 mg/L, and at a bed height of 8 cm. The maximal total phosphate adsorbed onto the coal material’s surface was 243 mg/kg adsorbent. The Adams–Bohart model depicted the experimental breakthrough curve well, and overall performed better than the Thomas and Yoon–Nelson models did, with correlation values (R^2^) ranging from 0.92 to 0.98. Lastly, leftover coal could be used in the purification of phosphorus-laden water, and the Adams–Bohart model can be employed to design filter units at a technical scale.

## 1. Introduction

Increasing concern about the effect of phosphate released from different natural and human activities has resulted in more stringent environmental policies in recent years. Phosphate is one of the main nutrients for plants and aquatic lives, and is, in turn, primarily responsible for the eutrophication of water [1,2,3,4,5,6]. The eutrophication of water bodies due to phosphate discharges is a challenging issue for industrialized regions [7,8]. Domestic activities, detergent-making industries, and mining companies are the primary sources for phosphate discharged to water bodies. The uncontrolled use of fertilizers also releases phosphate and affects nearby water streams due to the runoff from agricultural activities [9,10]. Water pollution by phosphate is tremendously increasing, and demand for the removal of excess phosphate from water bodies is thereby also increasing [11].

There are numerous methods to remove phosphate from water, including chemical precipitation, biological treatment, physical process, coagulation, and adsorption [12,13,14,15]. Nevertheless, most of them, with the exception of adsorption, show drawbacks due to the high sludge production, the complexity of the process, and high operational costs compared with adsorption methods [16,17]. Adsorption has received immense interest due to its high removal efficiency, simple operation, better cost effectiveness, less or no sludge production, and invulnerability to coexisting pollutants [18,19,20]. The application of low-cost and locally available materials for phosphate removal was widely investigated during this decade, such as with modified biochar [20], steel slag [21], volcanic rock [22], alkaline Tunisian soil [23], chitosan composite [24], alum sludge [25], and furnace slags [26].

Numerous studies were applied as adsorption methods and to remove phosphate from water by using batch experiments with different low-cost adsorbents [24,27,28,29]. At the industrial scheme, however, it was difficult to remove phosphate from a large amount of a water solution at the industrial level using batch adsorption [30], so continuous-flow fixed-bed column adsorption is versatile in the removal of phosphate from large amounts of a water solution using low-cost adsorbents [12].

In the present study, phosphate removal potential was examined in a fixed-bed column by using leftover coal material. The same authors verified the application of leftover-coal [31] and volcanic-rock [22] material as potential adsorbents for the recovery of phosphate from an aqueous solution through classical slurry batch experiments. However, batch-adsorption data are inconvenient for large-scale volumes due to the overestimation of sorption capacities [32]. In order to obtain convincing results, fixed-bed column adsorption is more realistic and popular in water treatment plants due to its continuous, high-yield, easy, and economical operation, and the ability to be scaled up from the laboratory scale [33]. Furthermore, few or no studies have reported on the phosphate adsorption capacity of leftover coal via continuous-flow fixed-bed column methods. Therefore, the main objective of this study is to: (i) investigate the application of fixed-bed column for the removal of phosphate using leftover coal material, (ii) study the effects of operational parameters on removal capacity by using breakthrough-curve analysis, and (iii) correlate experimental data with theoretical breakthrough-curve models to predict overall adsorption behaviors.

## 2. Materials and Methods

### 2.1. Adsorbent Preparation and Characterization

The coal material was obtained from Yayu coal mining, as presented in our previous study [31]. Prior to the experiments, the coal material was washed several times using deionized water to remove dust, dirt, and adhering particles from the surface of the adsorbent, and then dried for 24 h at 105 °C in an oven dryer. The well-dried materials were ground into a powder form. The ground powder was sieved to the size of 0.075–0.425 mm. The well-prepared adsorbent materials were labeled and stored in desiccator until the following experiments.

Proximate and ultimate analyses of the adsorbent material were performed according to our previous research [31]. The pH zero point charge (pHzpc) of the adsorbent material was measured according to [34] by using the solid-addition method. The organic matter, moisture content, and pHzpc of the material were measured to be 28.5%, 0.93%, and 4.6, respectively.

**Figure 1:**
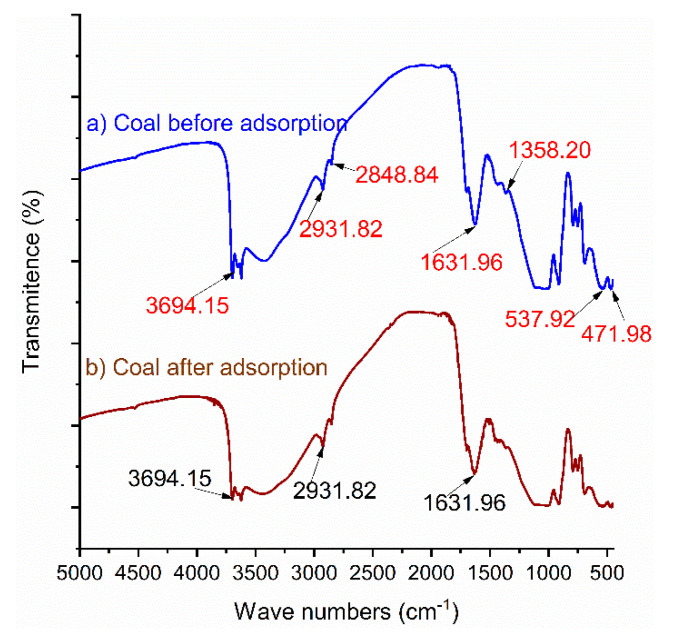
FT-IR results for leftover coal material (**a**) before and (**b**) after adsorption.

The functional groups of the adsorbent material were recorded using an FTIR machine (PerkinElmer, FT-IR spectrometer—Spectrum two) in the mid infrared region of 450–5000 cm^−1^ with a spectral resolution of 2 cm^−1^. First, the pellet was prepared from dried sample materials by the proper mixing of potassium bromide (KBr) with a 1:9 adsorbent-to-KBr ratio [35] and ground to a very fine size. The well-mixed material was pelletized using a pressure corporation machine, and the pellets were then measured accordingly [36]; the Fourier transform infrared (FT-IR) of the adsorbent material is shown in Figure 1.

**Figure 2:**
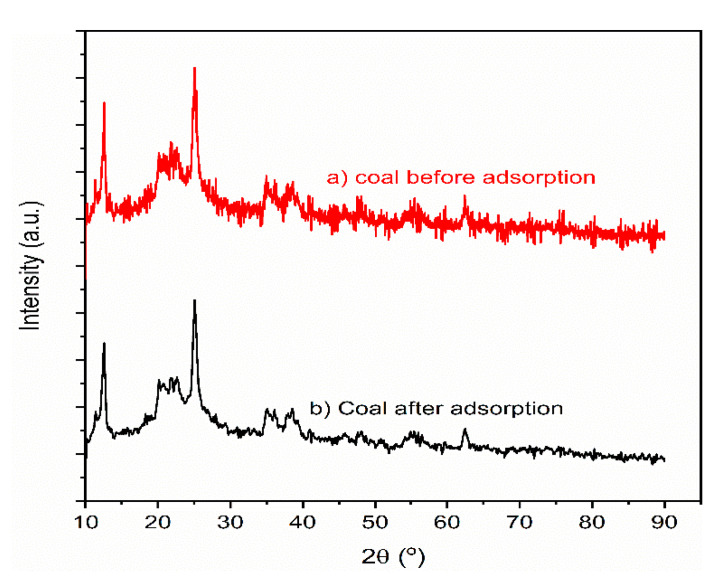
XDR patterns of leftover coal material (**a**) before and (**b**) after adsorption.

X-ray diffraction (XRD) analysis was performed to observe the crystal structure and mineral composition of the adsorbents. The XRD patterns of the leftover coal were recorded using an XRD apparatus (XRD-7000, SHIMADZU Corporation, Japan). Before XRD analysis, the leftover coal material was washed, dried in an oven to the appropriate temperature, and milled and sifted through a 75-micrometer sieve to obtain uniform and homogeneous particles. Then, XRD analysis was recorded with CuKα as the source radiation at wave length of 1.4 nm, at 40 kV and 30 mA (Figure 2). All employed chemicals and reagents in this study were of analytical grade unless otherwise specified.

### 2.2. Phosphate Adsorption in a Fixed Bed Column

The fixed-bed column adsorption experiments were conducted using a Pyrex glass column of 130 mm height and 26 mm diameter. The column was packed with a known amount of adsorbent material (leftover coal) to achieve the intended bed heights (5 to 8 cm), and then caped at the top and at the bottom with glass wool to avoid bed height changes due to the loss of some adsorbents and to prevent adsorbent wash out. Artificial phosphate-laden wastewater was synthesized by previously diluting phosphate stock solution with a concentration of 1000 mg/L to the working solution at concentrations of 10 to 25 mg/L using distilled water, and the pH of each working solution was adjusted to 3.5 using HCl and NaOH by pH meter according to our previous works [22,31]. Depending on the pH of the working solution, phosphorus can be present in the solution in the form of H_3_PO_4_, H_2_PO_4_^−^, HPO_4_^2−^, and PO_4_^−3^. At the working pH of 3.5, dihydrophosphate ion (H_2_PO_4_^–^) species was dominating, which can be seen from the repartition or speciation diagram of phosphate ion [37].

Prior to the experiments, the filled and packed column was flushed upward by deionized water until steady flow conditions were established [38]. The working solution then pumped up through the column with different flow rates (1 to 2 mL/min) using a peristaltic pump (MS-REGLO, Labortechnik-Analytic, Zurich, Switzerland). In order to obtain the breakthrough curves, the effluent was collected at a predetermined interval of time. Lastly, equilibrium phosphate concentration was measured by continuous flow analyzer (AA3 from Seal analytical GmbH, Norderstedt, Germany).

### 2.3. Theory and Column Data Evaluation

The obtained data from the fixed-bed column studies were used to estimate the breakthrough curve by plotting the C_t_/C_o_ (the ratio of effluent phosphate concentration at time t to influent phosphate concentration) vs. time. The breakthrough curve, therefore, could be calculated at breakthrough time (t_b_) at which the C_t_/C_o_ = 90% and exhaustion time (t_e_) at which Ct/Co = 10%, defined as C_t_/C_o_ = 0.9 and C_t_/C_o_ = 0.1, respectively. The breakthrough curve and its shape are the main characteristics that are used to investigate the dynamic response of the adsorption system [39]. For a desired initial phosphate concentration and flow rate, the total amount of phosphate retained by the column, q_total_ (mg) could be calculated from the area under the breakthrough curve (Equation (1)), while the mass of phosphate adsorbed per mass of adsorbent at equilibrium (qe (mg/g)) was calculated from Equation (2) [40].

(1) $$q_{\mathrm{total}}=\frac{{\mathrm{QC}}_{o}}{1000}\int_{t=0}^{t=\mathrm{total}}\left(1-\frac{C_{t}}{C_{o}}\right)\mathrm{dt}\;$$

(2) $$q_{e}=\frac{q_{\mathrm{total}}}{m}\;$$

where Q is the volumetric flow rate (mL/min), and m is mass of adsorbent packed within the column (g).

The mass transfer zone (MTZ) is defined as the active region of the coal material where the adsorption of phosphate takes place and can be defined as Equation (3) [41,42].

(3) $$\mathrm{MTZ}=H_{b}\left(\frac{t_{e}-t_{b}}{t_{e}}\right)\;$$

where H_b_ is total bed height (cm), and t_b_ and t_e_ are the breakthrough time and exhaustion time (min), respectively, which can be obtained from the area under the curves (Equations (4) and (5)) [43,44,45].

(4) $$t_{b}=\overset{t_{b}}{\underset{0}{\int }}\left(1-\frac{C_{t}}{C_{o}}\right)\mathrm{dt}\;$$

(5) $$t_{e}=\int_{0}^{t_{\mathrm{total}}}(1-\frac{C_{t}}{C_{o}})\mathrm{dt}$$

Empty bed contact time (EBCT) is the time of contact between the water phase and the adsorbent (Equation (6)), and it measures critical depth and contact time for the adsorbent [23].

(6) $$\;\mathrm{EBCT}=\frac{V_{b}}{Q}$$

where V_b_ is adsorbent bed volume (mL), which can be described as ($V_{b}={\pi r}^{2}H_{b}$, H_b_ is the corresponding bed height in cm, r is the inner radius of the bed column tube), and Q is volumetric flow rate (mL/min). The total amount of phosphate (P_total_, mg) entering the bed can be used to determine the removal efficiency of the column, and can be calculated according to Equation (7) [9,35,46].

(7) $$P_{\mathrm{total}}=\frac{{\mathrm{QC}}_{o}t_{\mathrm{total}}}{1000}$$

where t_total_ (min) is the operation time for the saturation of the adsorbate. Effluent volume Ve (mL) can also be calculated from the product of flow rate and total time, where influent volume V_i_ is obtained from the product of flow rate and break though time [19,47].

### 2.4. Theoretical Breakthrough-Curve Models

Several theoretical adsorption models have been used to predict the dynamic adsorption behavior of the leftover coal in a fixed-bed column. Therefore, in this work, three of most commonly used mathematical models, the Thomas, Adams–Bohart, and Yoon–Nelson models, were applied.

Thomas model: It is the most widely used theoretical model to evaluate column performance and predict concentration–time profile of the whole breakthrough curve. The model assumes that adsorption is limited by the mass transfer at the interface rather than chemical interactions of the molecules; experimental data obey the Langmuir isotherm and the second-order reversible kinetic model [10,20]. The linear form of the Thomas model can be expressed using Equation (8).

(8) $$\ln(\frac{C_{o}}{C_{t}}-1)=\frac{K_{\mathrm{Th}}q_{\mathrm{Th}}m}{Q}-{\;K}_{\mathrm{Th}}C_{o}t\;$$

where C_o_ and C_t_ are influent and effluent phosphate concentration (mg/L), respectively, K_Th_ (mL/min.mg) is the Thomas rate constant, q_Th_ (mg/g) is predicted adsorption bed capacity, m (g) mass of adsorbent packed within the column, and Q (mL/min) is flow rate. From the plot of $\ln(\frac{C_{o}}{C_{t}}-1)$ vs. t, the values of K_Th_ and q_Th_ can be determined using the slope and intercept, respectively.

Adams–Bohart model: The bed depth service time (BDST) is the other linearized form of Adams–Bohart (A–B) model expression, commonly applied to illustrate the relationship between service time and bed depth at fixed-bed column study [48,49]. The A–B model generally predicts a linear relationship between bed height and the time required to reach breakthrough time [50]. The linear form of A–B model can be expressed by Equation (9) [19,51].

(9) $$\ln(\frac{C_{t}}{C_{o}})={\;K}_{\mathrm{AB}}C_{o}t\;-{\;K}_{\mathrm{AB}}N_{o}\left(\frac{H_{b}}{U}\right)\;$$

where K_AB_ (L/mg min) is the Adams–Bohart rate constant, N_o_ (mg/L) is saturation concentration in column (adsorption capacity per unit volume), H_b_ (cm) is the column bed height, and U (cm/min) is linear velocity and is calculated by dividing Q (mL/min) by the cross sectional area (cm^2^) of the bed ($U=\frac{Q}{A}$). This model is more appropriate for describing the initial parts of the adsorption breakthrough curve at which Ct/Co = 0–0.5 [10,41]. The values of K_AB_ and No are determined from the linear plot of $\ln(\frac{C_{t}}{C_{o}})\;\mathrm{vs}.\;$t, which are equivalent to the slope and intercept of the linear plot, respectively [50].

Yoon–Nelson Model: This theoretical model is based on the assumption that the rate of adsorption for each adsorbate is proportion to the rate of decrease in adsorption. The model does not require explicitly elaborated information about the characteristics of adsorbate and its type [52]. It rather predicts 50% of the breakthrough time and simulate the column data obtained from single adsorbate system [15]. The linear form of the model can be expressed by Equation (10) [48].

(10) $$\ln(\frac{C_{t}}{C_{o}-C_{t}})={\;K}_{\mathrm{YN}}t-{\;\tau K}_{\mathrm{YN}}\;$$

where K_YN_ (min^−1^) is the Yoon–Nelson rate constant, τ (min) is the required time for 50% of the phosphate breakthrough and t (min) is the running time. The values of K_YN_ and τ can be obtained from the linear plot of $\ln(\frac{C_{t}}{C_{o}-C_{t}})$ vs. t using the slope and intercept respectively.

## 3. Results and Discussions

### 3.1. Adsorbent Characterization

The FT-IR spectrum of the adsorbent material (leftover coal) at wavelengths ranging from 5000 to 450 cm^−1^ is shown in Figure 1. Five major bands were identified for the FT-IR spectrum of the coal material, from which a strong and broad band was formed due the stretching vibration of–OH functional groups at the wave number of 3694.15 cm^−1^; the band around 1631.96 cm^−1^ indicated the bending vibration of –NH in NH_2_ for the FT-IR spectrum of coal materials [53,54]. The band obtained at the wave number of 2931.82 cm^−1^ indicated stretching vibrations of C–H [55,56]. The peak observed in FT-IR spectrum band at 1358.20 cm^−1^ for coal material is characteristic of the SiO_4_^−2^ group, and is caused by the symmetric stretching vibration of Si–O–Si and the stretching vibration of –CO in –COH [53,54,57]. The small peaks shown at the bands of 537.92 and 471.98 cm^−1^ in the FT-IR spectrum of the leftover coal material belong to the bending vibration of Si–O–Si bonds [43,58]. As shown in Figure 1b, after column adsorption, some weak bands occurred at the band wavelengths of 537 and 471 cm^−1^. This shows the adsorption of phosphate onto the surface of the adsorbent [46,59].

The XRD pattern of the leftover coal material before and after column adsorption is presented in Figure 2. A diffraction peak at 2θ = 12.6 degree is associated with kaolinite (Al_2_O_3_2SiO_2_·2H_2_O), and the typical peak obtained at 2θ = 25.4 degrees was induced by quartz (SiO_2_). The small peak at 2θ = 35 degrees belongs to goethite, consisting of iron (III) oxide–hydroxide [27,60]. Data for coal material obtained from XRD patters revealed the dominance of Si in coal material, which agrees with obtained SEM/EDX data from our previous work [31]. The crystalline size of the XRD pattern of the coal martial can be calculated from the Scherer equation as presented in Equation (11).

(11) $$D=\frac{K\lambda }{\beta \cos\;\theta }\;$$

where *D* is the average crystalline size (nm), K crystal shape factor or Scherer constant (0.68 to 2.08 and 0.94 for spherical crystallites with cubic symmetry) [61], λ is X-ray wavelength of radiation which is for Cukα = 1.4 Å, β peak breadth or line broadening at full width at half maximum (FWHM) in radian, and θ is equal to ½ of the 2θ position of the peak. The crystalline of the leftover coal material was 6.2 nm. However, the XRD peaks of the coal material showed amorphous rather than crystalline surface; the more amorphous the surface was, the higher the adsorption capacity [27,62].

### 3.2. Effects of Operational Parameters

#### 3.2.1. Effects of Adsorbent Bed Height

**Figure 3:**
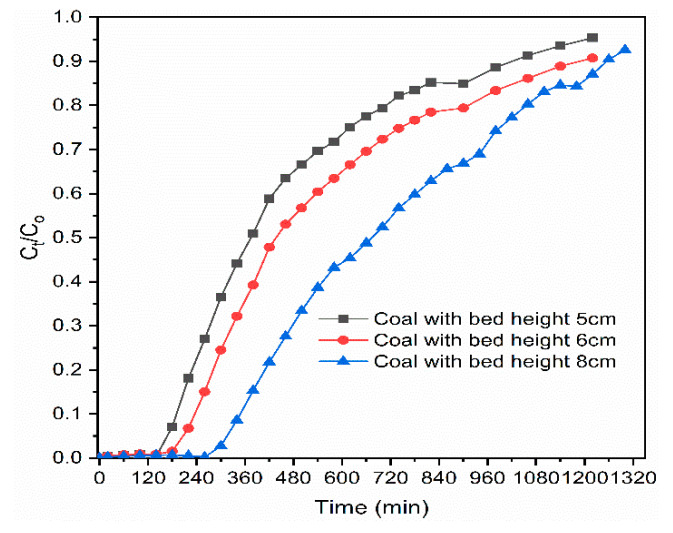
Effect of adsorbent bed height on phosphate breakthrough.

The effect of bed heights on the breakthrough curves of column adsorption were studied using bed heights of 5, 6, and 8 cm with a constant influent phosphate concentration of 10 mg/L and at a fixed flow rate of 1 mL/min. Figure 3 shows the related breakthrough curves with different bed heights. The removal efficiency of the phosphate was proportional to the proposed bed height. In all cases, breakthrough times were extended from 190 to 235 min, and then to 348 min, with increasing bed height from 5 to 8 cm. Subsequently, the influent volume of phosphate solution treated at the breakthrough time (V_i_) and at exhaustion time (V_e_) increased from 190 to 348 mL and from 273 to 381 mL, respectively, for longer bed heights as compared with for the shorter one (Table 1). This indicates that the shorter beds saturated faster than the longer one did because the longer bed heights need longer to become saturated [18]. The increase in Vi and Ve was probably due to the high contact time between phosphate ions and adsorbent [19]. Furthermore, the longer bed height (8 cm) used in this study had higher phosphate adsorption capacity (190.7 mg/kg) than that of 6 cm (178 mg/kg) and 5 cm (163 mg/kg) bed height. Therefore, the longer the bed height was, the longer the time taken was to reach complete exhaustion time due to broadened mass transfer zone for phosphate adsorption [47,48].

#### 3.2.2. Effects of Influent Concentrations

**Figure 4:**
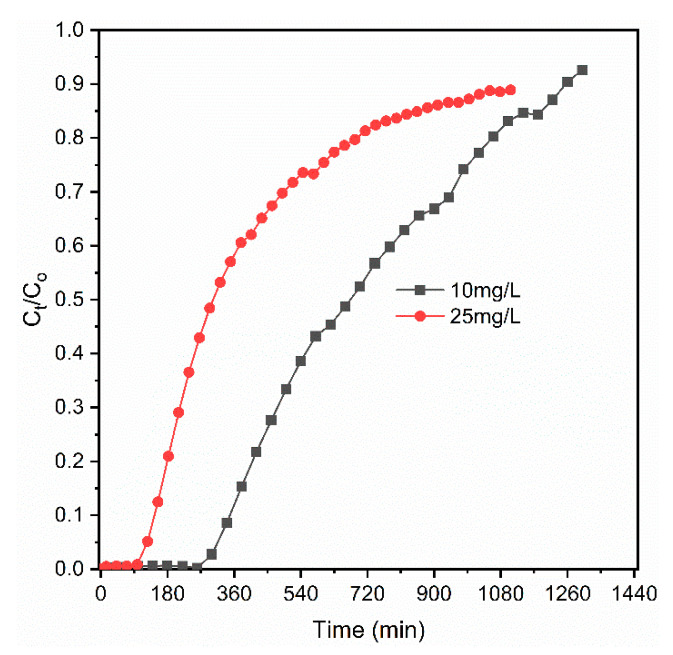
Effect of influent concentration on phosphate behaviors.

The effect of initial phosphate concentration on the performance of the column was studied by varying the inlet concentration from 10 to 25 mg/L while the same adsorbent bed height of 8 cm and flow rate of 1 mL/min were used. Figure 4 shows that a very fast breakthrough time occurred at the inlet concentration of 25 mg/L. At a higher influent concentration of the phosphate, the quick fill of the binding sites of the adsorbent material was observed [41,63]. As the influent phosphate concentration increased from 10 to 25 mg/L, exhaustion time also decreased from 348 to 144 min. The lower phosphate concentration caused the slower diffusion of the phosphate than that of the higher concentration onto the surface of the coal material due to the lower mass transfer coefficient, and contributed to the longer breakthrough time and exhaustion time [18,41]. Equally, the higher the influent concentration was, the higher the concentration gradient and the lower the mass resistance were with shorter breakthrough time and exhaustion time. Similar tendencies were reported by [64] in the case of removing phosphate from an aqueous solution using zirconium-loaded okara. Nevertheless, the increase in influent concentration increased the phosphate removal capacity (from 190 to 243 mg/kg), but the total influent volume (Vi) of the treated solution was decreased from 348 to 144 mL. Utilizing the lower influent phosphate concentration is preferable to a higher concentration when the treatment of a larger volume is prioritized. Calculated parameters for different concentrations are presented in Table 1.

#### 3.2.3. Effects of Influent Flow Rate

**Figure 5:**
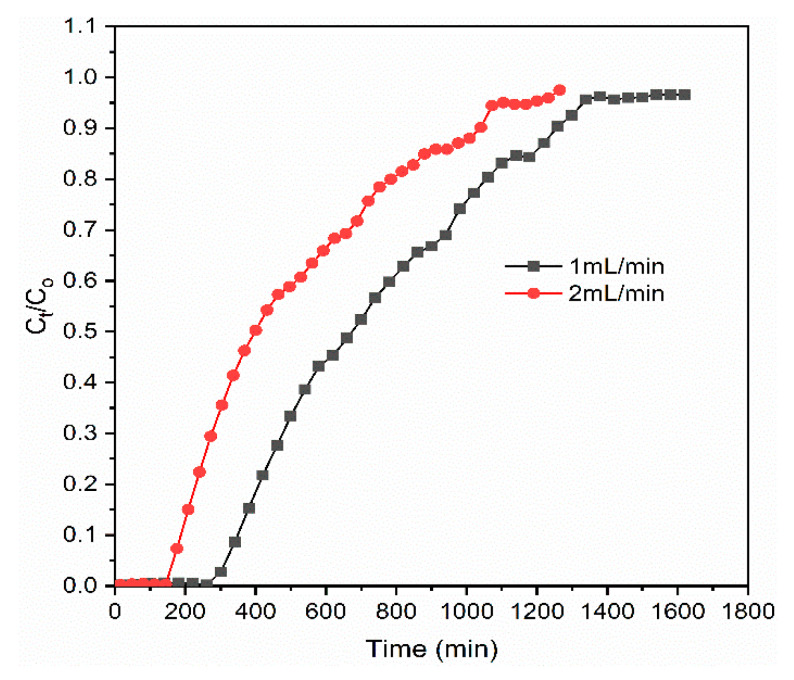
Effect of influent flow rate on phosphate breakthrough.

The breakthrough curves at different flow rates (1 and 2 mL/min) using influent phosphate concentration of 10 mg/L and adsorbent bed height of 8 cm are presented in Figure 5. The breakthrough time and exhaustion time of the adsorbent material appeared significantly faster, which was related to the mass transfer process when the used flow rates were increased from 1 to 2 mL/min. In this case, the breakthrough time decreased from 348 to 187 min, and exhaustion time decreased from 381 to 313 min for the increase in flow rate from 1 to 2 mL/min. Another study reported a very fast breakthrough time (<40 min) for the removal of phosphate from a solution using andosol bagasse mixtures at the influent solution flow rate of 4 mL/min in 1.8 cm bed height [19]. According to Chittoo and Sutherland (2020) [65], increasing the flow rate may reduce the resident time of the adsorbate to diffuse into the pores of the adsorbent materials; thus, the adsorbates predominately interact with surface functional groups. At lower flow rates, adsorbates have enough time to interact with the surface of the adsorbents, and additional external mass transfer and intraparticle diffusion are thus enhanced [66], where, at the higher flow rate, the adsorbate solution leaves the column before reaching the equilibrium point [67]. The adsorption parameters for varying bed depths, concentrations, and flow rates are presented in Table 1.

### 3.3. Prediction of Breakthrough Curves Using Adsorption Models

Predicting fixed-bed column parameters in continuous flow analysis is very important for both laboratory- and industrial-scale processes [15]. The breakthrough curve is a plot of ratios of effluent to influent concentrations versus running time, and its prediction provides the mechanisms and generally any changes in the adsorption processes. In the present study, three theoretical models, namely, the Thomas, Adams–Bohart, and Yoon–Nelson models, were applied to determine the best model for predicting the dynamic behaviors of the models [68,69].

The Thomas model is frequently used to determine adsorption capacity of the adsorbent. The rate constant, adsorption capacity, and other parameters were obtained from the linear plot of Equation (8) and numerical values are presented in Table 2. Table 2 shows that K_Th_ values increased from 1.67 × 10^2^ to 2.83 × 10^2^ mL/min mg, while the values of q_Th_ decreased from 328 to 203 mg/kg with increasing adsorbent bed height from 5 to 8 cm. However, other researchers reported a decreasing value of K_Th_ and increase in q_Th_ with increasing bed depth. This is may have been due to the experimental measuring and applying different adsorbents. On the other hand, the values of K_Th_ decreased with increasing influent phosphate concentration and flow rate. These values can be illustrated with the driving force due to the increase in concentration gradients and flow rate as reported in other studies [19,32]. Regression values (R^2^) from the Thomas model ranged from 0.84 to 0.94, which illustrated a worse fit than that of other models.

**Figure 6:**
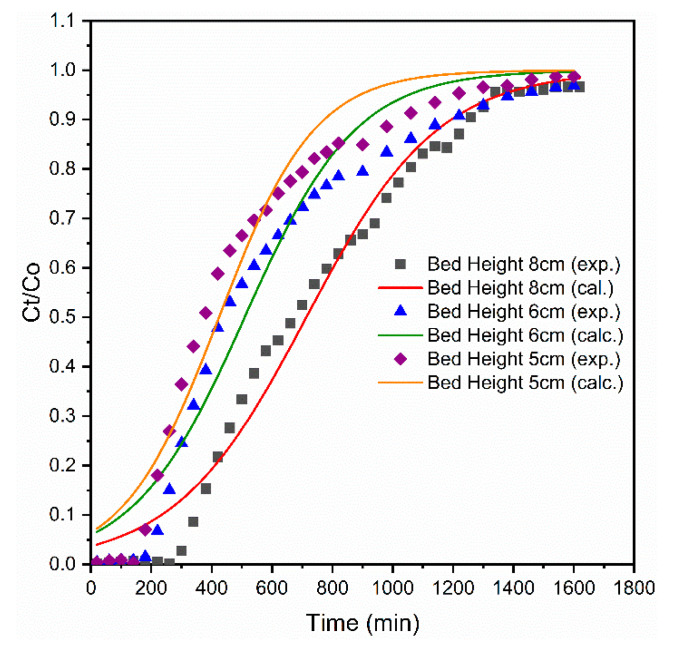
Experimental and calculated breakthrough curve for Adams–Bohart model at different values of adsorbent bed height.

**Figure 7:**
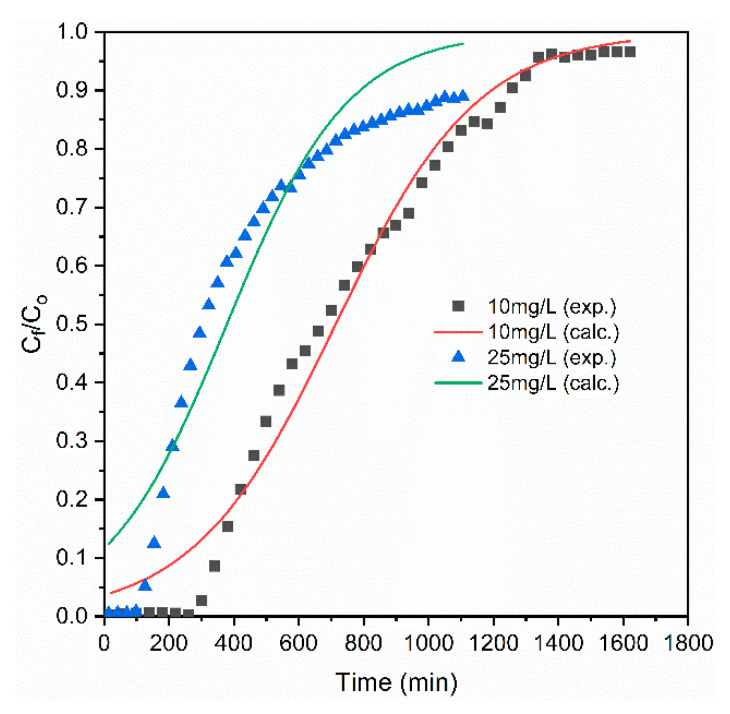
Experimental and calculated breakthrough curve values for Adams–Bohart model at different values of influent phosphate concentration.

The calculated data for the Adams–Bohart model are presented in Table 2. K_AB_ values increased with increasing adsorbent bed height, but decreased with increasing influent phosphate concentration and flow rate. The values of No decreased with increasing bed height and influent phosphate concentration, but increased with increasing flow rate. The calculated and experimental data for the A–B model using initial phosphate concentration and adsorbent bed height are illustrated in Figure 6 and Figure 7, where the R^2^ values of A–B model ranged in between 0.92 to 0.98, which provided a better fit than that of the Thomas and Y–N models.

The Yoon-Nelson (N–Y) rate constant, K_YN_, and time required for 50% breakthrough τ values at different operational parameters are presented in Table 2. The values of both K_YN_ and τ were decreased with increasing flow rate and influent phosphate concentration. The fitness of the N–Y model was evaluated using correlation values (R^2^) ranging from 0.90 to 0.94. The Y–N model described the behavior of sorption of phosphate better than the Thomas model did under different experimental conditions. However, the Adams–Bohart model described the adsorption of the phosphate in the column better than the Thomas and Y–N models did at different operational conditions.

## 4. Conclusions

This study was conducted to test leftover coal as a material for the removal of phosphate from solutions by adsorption using continuous-flow fixed-bed column experiments. The adsorption performance and the breakthrough-curve characteristics of the column were influenced by influent phosphate concentration, adsorbent bed height, and the influent flow rate of the solution. The increase in bed height significantly improved the performance of the column by increasing the breakthrough and exhaustion times of the process, and increased the volume of water that could be treated. On the other hand, the increase in initial phosphate concentration and influent flow rate reduced the breakthrough and exhaustion times. This was due to the fast saturation of the surface of the adsorbent with phosphate ions and a lower resident time of the adsorbate. Adam–Bohart model fitted the experimental data well, and performed better than the Thomas and Yoon–Nelson adsorption models did. Overall, the results of this study suggest that phosphate removal using leftover coal material is a promising low-cost technology for the sustainable control of excess phosphate in water. However, additional testing of the adsorbent using surface modification and applying real sample water with competitive anions are required for final conclusions.

## Figures and Tables

**Table 1:** Obtained parameters from breakthrough curves for phosphate adsorption onto leftover coal material with different bed heights, initial phosphate concentrations, and flow rates.

C_o_ (mg/L)	H_b_ (cm)	Q (mL/min)	V_i_ (mL)	t_b_ (min)	t_e_ (min)	EBCT (min)	MTZ (cm)	q_total_ (mg)	q_e_ (mg/kg)	V_e_ (mL)
10	8	1	348.39	348.39	381.37	607.79	5.78	5.72	190.70	381.37
10	8	2	374.06	187.03	313.39	303.89	6.56	6.27	208.90	626.78
10	5	1	190.68	190.68	273.21	257.33	3.96	4.10	163.90	273.21
10	6	1	235.73	235.73	297	357.81	4.59	4.46	178.20	297
10	8	1	348.39	348.39	381.37	607.79	5.78	5.72	190.70	381.37
10	8	1	348.39	348.39	381.37	607.79	5.78	5.72	190.70	381.37
25	8	1	144.45	144.45	291.92	607.79	6.96	7.30	243.20	291.79

V_i_ = influent volume; V_e_ = exhaustion/effluent volume.

**Table 2:** Parameters for Thomas, Adam–Bohart, and Yoon–Nelson models at different parameters.

Exp. Parameters				Thomas			Adams–Bohart			Yoon–Nelson		
	Co (mg/L)	H_b_ (cm)	Q (mL/min)	K_Th_ × 10^2^ (mL/min.mg)	q_Th_ (mg/g)	R^2^	K_AB_ × 10^3^ (L/mg min)	N_o_ × 10^3^ (mg/L)	R^2^	K_YN_ (min^−1^)	τ(min)	R^2^
**Q (mL/min)**	10	8	1	2.830	0.215	0.87	0.188	1.690	0.98	2.831	6.453	0.90
	10	8	2	2.610	0.203	0.92	0.172	3.217	0.92	2.613	6.079	0.92
**H_b_ (cm)**	10	5	1	1.670	0.328	0.84	0.151	2.602	0.96	2.608	5.985	0.92
	10	6	1	2.430	0.253	0.93	0.155	2.188	0.98	2.430	6.174	0.93
	10	8	1	2.830	0.215	0.87	0.188	1.690	0.98	2.831	6.453	0.90
**C_o_ (mg/L)**	10	8	1	2.830	0.215	0.87	0.188	1.690	0.98	2.831	6.453	0.90
	25	8	1	2.120	0.197	0.94	0.143	1.578	0.92	2.121	5.908	0.94

## Data Availability

The data used in this study are available from the first author upon request.
